# Beech and silver fir’s response along the Balkan’s latitudinal gradient

**DOI:** 10.1038/s41598-019-52670-z

**Published:** 2019-11-07

**Authors:** Matjaž Čater, Tom Levanič

**Affiliations:** Slovenian Forestry Institute, Department of Yield and Silviculture, Večna pot 2, 1000 Ljubljana, Slovenia

**Keywords:** Climate-change ecology, Forest ecology, Light responses

## Abstract

At the 1000 km geographical distance in Dinaric montane forests of silver fir (*Abies alba* Mill.) and European beech (*Fagus sylvatica* L.), the tree response from the north-western sites towards southern, warmer and dryer sites was performed during three consecutive growing seasons (2011, 2012 and 2013). On eleven permanent plots, positioned in uneven-aged beech and fir forests above 800 m along the geographical gradient, the physiological and morphological response to light intensity were measured in predefined light categories based on the analysis of hemispherical photos. Radial growth was analysed on all plots and compared to precipitation, temperature and two drought indexes. Analysis showed a decrease in the cumulative precipitation and no change in temperature between plots. Beech was most efficient in the open area light conditions, while fir proved most efficient under shelter. Physiological response for beech increased towards SE and reached its maximal values in the middle of transect, while fir’s response decreased from the NW towards SE. Tendency to plagiotropic growth decreased from NW to SE in both species. Growth response to climatic parameters is weak, stronger in fir than in beech and decreasing towards SE.

## Introduction

Montane forests of silver fir (*Abies alba* Mill.) and European beech (*Fagus sylvatica* L.) in the Dinaric region as the largest contiguous forest area in Central Europe^1^ harbour several protected areas (e.g. National parks, Natura 2000) and habitats for many endemic and endangered species. As a long-lived tree, fir is considered a significant ecological and functional species^2^, which stabilizes soils and retains water, and is less susceptible to windthrow and snow or ice breakage than Norway spruce^3^. Fir is also considered a fundamental species for maintaining high biodiversity in forest ecosystems because of its shade tolerance, ability to survive long periods in the understory and to respond when light conditions become more favourable, plasticity to environmental conditions and ability to coexist with many tree species^3,4^. Both fir and beech, as main coexisting species in montane, mixed-species forests, are shade tolerant, and could thrive under conditions of deep shade for longer time periods^5^. Fir is late successional tree, more sensible to water deficits than beech^6^ on drier sites^7^.

Despite the high degree of forest naturalness, fir’s regression is one of the major concerns in the whole region. It was observed already in the 1930s and 1950s, and was attributed mostly to climatic extremes coupled with bark beetle calamities e.g.^8,9^. Later, polluted air contributed to fir’s decline^10^, while its regeneration was exposed to overbrowsing in parts of the region^11^. Intensity of fir regression varied due to different combinations of causes of decline across the region^12^. Current size structure and regeneration characteristics indicate further regression of fir in the next decades. In the Mediterranean region, fir is more sensitive to drought and changes in the seasonal distribution of precipitation^13^ compared to non-native Norway spruce. It is a large tree important for site productivity, which forms many special habitats as a veteran or slow decomposing dead tree. Fir is economically much appreciated and the most important conifer tree species within the Dinaric region.

European forests are facing enormous threats from rapid global climate change (GCC) with increasing frequency and intensity of summer droughts; considerable uncertainties exist about plants potential to respond to future warming and declining moisture availability^14,15^. In the Mediterranean Basin, drought is the main limiting factor for tree growth^16^, where extreme events are expected to increase^17^ and lead to even higher soil moisture deficits during growing periods. Changes in the forest productivity^18^ and species distribution in many regions are likely to be expected^19^.

Current predictions of climate change impacts on plant demography rely on the association between species’ current geographical distribution and corresponding climate characteristics, invoking hypothetical constraints imposed by temperature and/or moisture availability extremes on one or more stages of a species’ life cycle^20^. The exact nature of such constraints is, however, unknown and lacking a sufficiently mechanistic basis^21,22^. To predict species responses to climate changes, physiological limits should be evaluated to obtain a complete representation of the fundamental niche of a species and then constrain it with biotic interaction and dispersal limitation effects^23,24^. In northern and western Europe, the increasing atmospheric CO_2_ content and higher temperatures are expected to result in favourable effects on forest growth and wood production, while increasing drought and disturbance risks are likely to outweigh positive trends in southern and eastern Europe.

Studies of tree response along the latitudinal gradient have been performed in boreal zones^25,26^, in eastern part of the Iberian Peninsula on black pine^19^, and across southern distribution limits in Spain, Italy and Romania on fir^27^. The dependency of tree growth on precipitation has increased during the last century and drought has experienced an upward trend after the 1950’s. The latitudinal progression of the radial growth decline and proportion of positive trends strongly support the rapid northward advance of Mediterranean climate caused by GCC and its effect on tree ecology and growth^27^.

Recent studies suggest a different response of fir along its distribution range^12^. Its disappearance from warmer and drier areas has been observed in Slovenia in fragmented forests, at the limit of its distribution^28^ and in southwestern Europe^27^, particularly in the Mediterranean region where fir’s decline is strongly related to increase in aridity^29^. Extreme weather events such as storms, droughts, frosts, lack of precipitation and an increase in average temperature will influence fir demography. A shift in fir distribution toward higher elevations and northwards is expected^30–32^. On the Balkan Peninsula, where different and well-expressed ecological factors intertwine at relatively short geographical distance (approx.1000 km)^33^, the studied tree response from the southern, warmer and dryer sites may serve as a most probable future prediction for the same species-response on currently less extreme sites.

Our aim was (1) to compare physiological and morphological responses of beech and fir along the defined 1000 km geographical gradient, (2) to evaluate differences in same light categories of both species between managed and old growth forest, and (3) to verify the connection between radial growth of adult stand with ecophysiological and morphological traits of both species along the gradient.

## Material and Methods

### Location and plot description

In one of the largest mountain regions in Europe, the Dinaric mountain chain extends from the southern edge of the Eastern Alps in Slovenia to the mountain massif in North Macedonia; it is bounded by the Adriatic Sea along its western border and the Pannonian Basin toward the east^34^. The main part of the range is formed of Mesozoic rocks, predominately of limestone and dolomite. The depth of the limestone and dolomite is unique, typically 1–3 km, with considerable local variation^34^.

Westerly winds blowing over the Adriatic Sea bring large amounts of humidity to higher elevations along the western side of the range. Precipitation throughout the upper elevation zone is relatively evenly distributed during the year, with snowpack often lasting up to six months^35^. Forest structure and composition in the region is strongly influenced by the interaction of the mountain relief, karst terrain, soils, and climatic gradient. Mountain forests above 800 m include mainly beech dominated forests and mixed uneven-aged forests dominated by varying amounts of beech, fir, and occasionally spruce. Large forested regions in the interior range have been left intact until the present days and have been managed with low intensity silvicultural systems for more than a century^36^, with several protected old-growth remnants scattered throughout the area.

**Figure 1:**
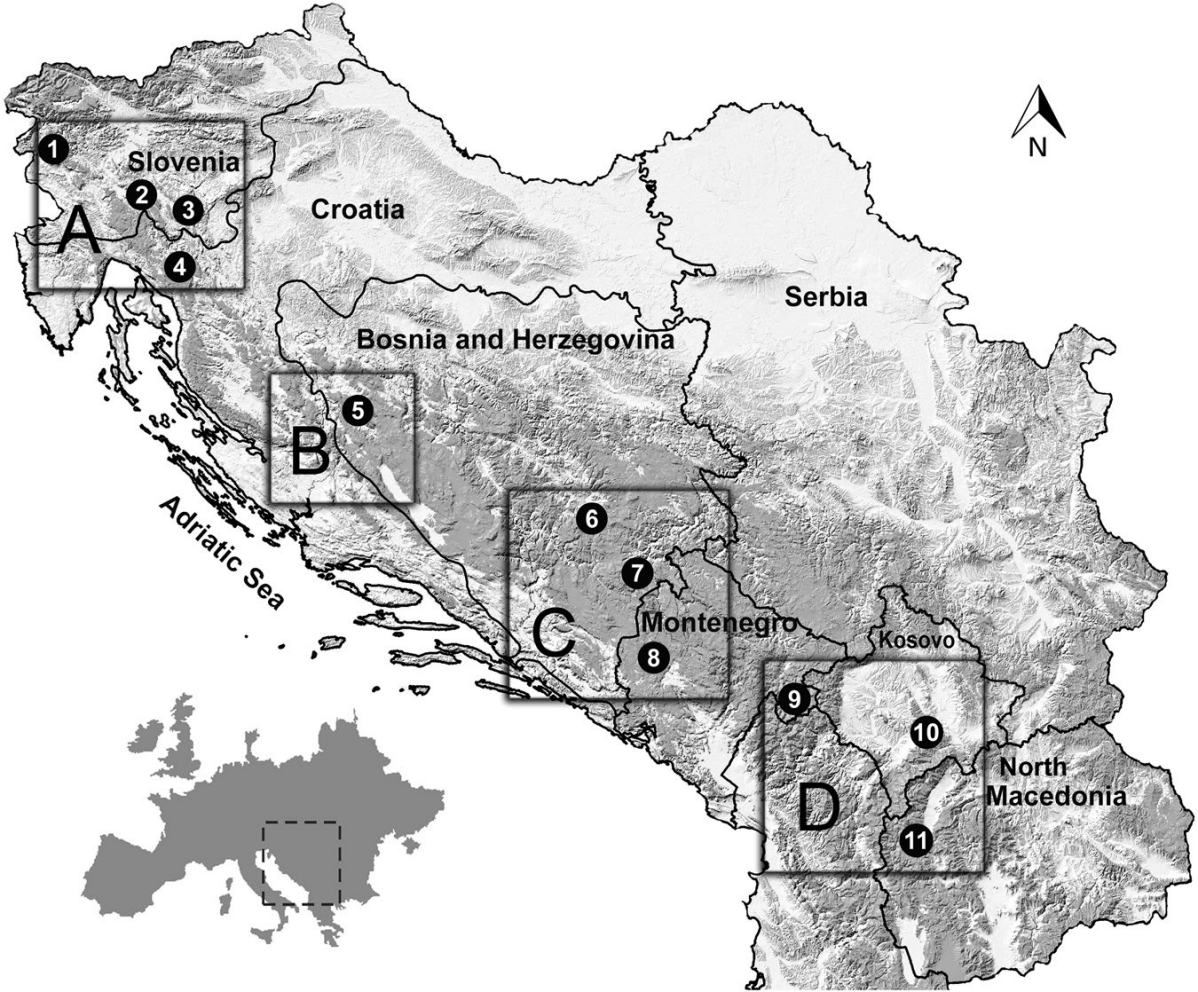
Research area and the location of plots. Elevations above 800 m are darker. Boxes (**A**–**D**) represent regions with extracted gridded meteorological data.

Eleven permanent plots were established in the optimally developed managed beech and fir adult forest stands, distributed from Slovenia from its furthest NW part over Croatia, Bosnia & Herzegovina (BiH) and Montenegro along the mountainous region of the Balkan Peninsula to North Macedonia on the SE part of the range. All selected plots were positioned at elevations above 800 m. Among selected locations three (plots No 3, 7 and 8) belong to the old growth reserves (Fig. 1, Table 1).

**Table 1:** Research plot characteristics; meteorological data was obtained from the Royal Netherlands Meteorological Institute ‘Climate Explorer’ web page (http://climexp.knmi.nl) for the last 1985–2015 period including total annual and April-September growing-season values.

Plot No	Region	Altitude ASL (m)	Latit. Deg (°)	Long. Deg (°)	Total annual precipitation (mm)	Average annual air T (°C)	Total Apr.-Sept. precipit. (mm)	Average April-Sept. air T (°C)
1	**A**	814	45°59′	13°45′	1619	11.3	863	15.2
2		807	45°41′	14°28′	1573	8.4	802	14.1
3		871	45°40′	15° 00′	1465	9.0	780	14.9
4		1190	45°16′“	14°48′	1108	9.3	616	14.9
5	**B**	928	44°18′	16°19′	1349	8.6	645	14.5
6	**C**	1204	43°44′	18°16′	1192	7.6	593	13.4
7		1313	43°19′	18°43′	1229	7.7	607	13.4
8		1408	42°59′	18°39′	1278	8.2	590	13.7
9	**D**	1402	42°33′	19°55′	1163	6.6	548	13.1
10		1410	42°15′	20°53′	850	8.6	418	14.6
11		1315	41°42′	20°44′	836	8.4	357	14.0

Plots 3, 7 and 8 represent old growth reserves.

On every plot, three categories of different light intensities were defined based on the analysis of hemispherical photos: under closed canopy with Indirect Site Factor (ISF) < 15%, at the forest edge (15% < ISF < 25%) and in the open ISF > 25%^37^. Assimilation response was measured in saplings of fir and beech in June and July during three consecutive growing seasons (2011, 2012 and 2013). Age of the trees varied between 5–12 years. In every light category and site, at least 12 trees were measured.

### Weather and climate

Data of mean monthly temperatures (°C) and total monthly precipitation were interpolated for the 0.5° grids including each sampled stand and corresponding to the CRU TS 4.01 dataset^38^. Climatic data were obtained from the Royal Netherlands Meteorological Institute ‘Climate Explorer’ web page (http://climexp.knmi.nl). For the comparison between A_max_, Φ, morphological response and climate (temperature and precipitation) data from the last 30-year average period (1985–2015) was used. For the long-term comparison between climate and tree growth, we defined four regions (A, B, C, D) where A is the northernmost and D the southernmost one. For each region we extracted gridded climate data for the mean monthly temperature and sum of monthly precipitation using CRU TS 4.01 dataset with 0.5 × 0.5-degree resolution from the KNMI web site (Fig. 1).

### Nitrogen content (N_tot_) and leaf mass per area (LMA)

Leaves and needles were sampled in the upper crown position of minimal 12 trees per light category and location, then cool-stored in airtight conditions. Same trees were used for the assimilation response measurements. Nitrogen concentration (N_tot_) [mg/g] was determined to compare macronutrient status (Leco CNS-2000 analyser)^39,40^ for open-, forest edge- and closed canopy-category below mature trees^41^. Fresh leaves were weighed and scanned for the leaf area. Leaves were dried at 105° for 24 hours until constant weight and weighed for the dry mass in the lab to provide leaf mass per area (LMA) [g/m^2^].

### Assimilation light response

The assimilation of beech and fir was measured on randomly distributed saplings along same light categories along three relative diffuse light (ISF) categories: of <15%, 15 to 25% and >25% defined by the hemispherical photos^41^. The hemispherical photos were taken at each sampling group of saplings separately, prior to further assimilation measurements. At least 12 young trees of the same height, unobstructed by their neighbours, were randomly chosen for light saturation measurements, performed during three sequential growing seasons (2011, 2012 and 2013) (*sensu*^41^). The light-response was measured with an LI-6400 portable system on at least four leaves/locations per tree, located in the upper third of the tree-crown.

Light saturation curves were established to compare the net assimilation (A_max_) in young beech and fir trees in the same light conditions. All assimilation measurements were performed in field at a constant temperature of the measurement block (20 °C), a CO_2_ concentration of 420 µmol/l, airflow 500 µmols^−1^ and different light intensities: 0, 50, 250, 600 and 1500 µmolm^−2^ s^−1^. Maximum assimilation (A_max_) rates for the light saturation curves were used for comparisons of responses between different light categories and plots.

The characteristic points of maximal quantum yield (Φ), defined as the maximal amount of fixed CO_2_ per amount of absorbed light quanta^40^, were established for each light category, species and plot, as described in Čater *et al*.^42^.

### Morphological response

Changes in crown morphology play an important role in the acclimation capacity of species to reduced light intensity under a mature canopy and in younger development stages. Crown morphological plasticity has been found to be especially important in shade-tolerant species^43^. Plagiotropism, result of deviation from the vertical axis, is the most unwanted effect with respect to future timber that increases with the degree of shade^44^.

To evaluate the morphological response of beech, the quotient between the length (l) and height of trees (h) was used, which increased in the case of plagiotropic growth. As young trees show slight deviation in growth from the vertical axis, which is not necessarily a function of plagiotropic growth, a wider threshold value of l/h ≥ 1.1 (110%) was chosen to separate plagiotropic from orthotropic growth. For fir maximal distance from the stem to the furthest branch, tip (d) was measured and compared with tree height (h) in all light conditions (ISF%); a threshold 2d/h ≥ 1.15 (115%) was used accordingly^37^. The limiting value of light was defined after measurements of the ratio after three consequential growing seasons (2011, 2012 and 2013). In the analysis, the values under same light-intensity conditions were compared between plots (how closed/open the mature stand was). Plagiotropic behaviour between different plots was assessed by comparison of data into an exponential-decay-3 parameter curve (1),

1 $$Y=A+B\ast {e}^{-Cx}$$

where *x* is the measured light (ISF%), *Y* is the quotient between the length (l) and the height of saplings (h) and *A, B* and *C* are the curve parameters.

### Growth response

Radial growth analysis for the last 100 years was conducted for adult beech and fir trees; two cores per tree from 15 adult dominant beech and firs on each location were taken, 660 in total. Each core was mounted and sanded to a high polish using sanding paper of progressively finer grit. The cores were then scanned using ATRICS system^45^ and annual radial increments measured to the nearest 0.01 mm using CooRecorder and CDendro software (www.cybis.se). We used the same software for quality control of measured tree-ring width sequences. Tree ring series were then visually and statistically cross-dated using PAST-5.

### Statistical analysis

Differences between same year (2011, 2012 and 2013) for the LMA, N_tot_, A_max_ and Φ were tested with the two-way ANOVA with species (beech and fir) and light (open, edge, canopy) as a dependent variable. Analyses of variance (ANOVA) and HSD Tuckey post hoc test were used after testing data to meet conditions of normality. Probability values of p < 0.05 (*), p < 0.01 (**) and p < 0.001 (***) were considered significant. Data analysis and correlation between measured variables was performed with Statistica data analysis software system (2011).

Tree-ring widths were standardised, using R software and “dpl” package^46^ with double detrending approach. First, a negative exponential curve was fitted to tree-ring width sequence to remove age trend, in the second step flexible, a 30-year cubic spline was fitted to the tree-ring width indices from the first step to remove any other disturbances and retain only climatic signal. Residual chronologies were calculated as the quotient between measured and fitted value and used in all climate-growth relationships, using R software and package “treeclim”^47^. Statistical relationship between meteorological months and residual chronologies for the 1902–2010 period from September prior to year of ring formation till September of current ring formation was analysed. We also ran a 31-year moving window correlation using only statistically significant combinations of meteorological data and tree-ring widths. Pointer years (PY) defined by Schweingruber *et al*.^48^ were calculated for every region.

## Results

### Climate

**Figure 2:**
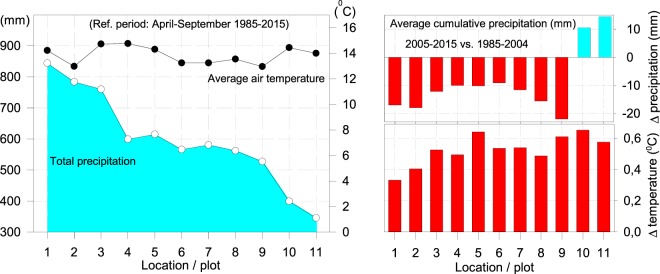
Average air temperature and total precipitation within the April-September (1985–2015) growing period on studied plots (left) and differences in average cumulative precipitation (mm) and in average air temperature (°C) between 1995–2004 and 2005–2015 windows during the same growing period (right).

On the studied range, climatic conditions in the SE differ from those in the NE: generally, the temperature in the southern part is higher, while the precipitation is lower. Sub-mediterranean mountainous climate prevails, characterized by high winter precipitation and markedly low summer precipitation^49^. Climatic condition for the last 30-year period indicate homogenous average temperature range on all studied plots within 12°–14 °C range and a significant decrease of annual amount of precipitation towards SE - from plot No.1 to plot No.11 (Fig. 2, left).

When comparing precipitation and temperature values of two different consecutive referential periods 1995–2004 and 2005–2015 within April-September, the drop of total precipitation and rise of average annual air temperature was confirmed (Fig. 2, right). On plots 1–9, evident decrease of precipitation between periods amounted to between 10–20 mm with the exception of plots 10 and 11, while the temperature rise ranged between 0.33°–0.65 °C on all studied plots.

### Nutrient status and leaf mass per area (LMA)

**Figure 3:**
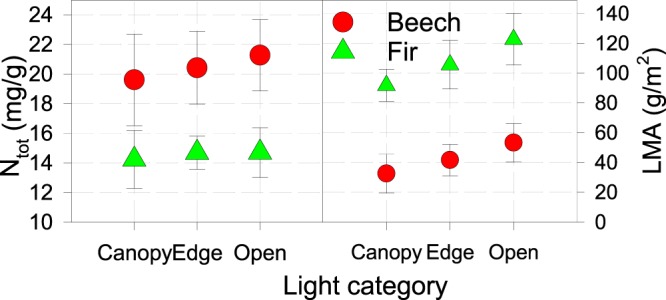
Foliar nitrogen and leaf mass per area; bars are standard errors.

The foliar nitrogen amount (N_tot_) for beech and fir was highest in the open and lowest under canopy conditions on all plots, without confirmed differences between same light categories on different plots and observed years. The content was found within optimal threshold values reported by Grassi and Bagnaresi^50^ or Mellert and Göttlein^51^. LMA increased from the shelter towards the open light category, with significant differences between shelter and open categories for both beech and fir (Fig. 3, Table 2).

**Table 2:** Results of ANOVA for N_tot_ and LMA of beech leaves and needles under canopy, edge and open light conditions.

Trait	Df 1; 2	Species		Df 1; 2	Shading		Df 1; 2	Species X Shading	
		F	p		F	p		F	p
N_tot_	1; 60	193.2	2e-16***	2; 60	3.594	0,0336*	2; 60	0.662	0.5197^NS^
LMA	1; 60	5859.9	2e-16***	2; 60	415.07	2e-16***	2; 60	46.03	7.6e-13***

### Assimilation response (A_max_) and quantum yield (Φ)

**Figure 4:**
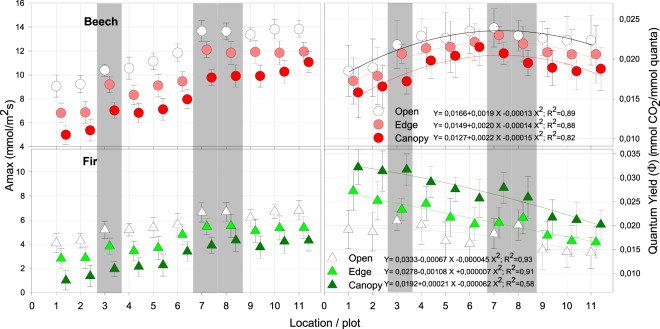
Assimilation (Amax) - left and Quantum yield (Φ) - right in three different light categories for beech and fir on studied plots. Shaded are the old growth reserves. Values of different light categories belonging to the same plot are shifted to avoid overlapping. Bars are standard errors of means.

For both beech and fir no difference was confirmed in A_max_ and Φ between observed years 2011–2012, 2012–2013 or 2011–2013, respectively. Evident increase in A_max_ in all light categories from NW to SE was confirmed for both species. Φ for beech increased from NW, reached peak in the middle of the studied transect and decreased towards SE, while maximal Φ for fir were in NW, followed by the evident decrease in all light categories towards SE (Fig. 4, right).

Differences in A_max_ and Φ between same light categories were significantly different for both beech and fir (Table 3).

**Table 3:** Results of ANOVA for A_max_ and Φ for beech and fir under canopy, edge and open light conditions.

Trait	Df 1; 2	Species		Df 1; 2	Shading		Df 1; 2	Species X Shading	
		F	p		F	p		F	p
A_max_	1; 522	1454.27	2e-16***	2; 522	1352.31	2e-16***	2; 522	89.53	2e-16***
Φ	1; 522	73.0	2e-16***	2; 522	231.0	2e-16***	2; 522	775.4	2e-16***

Different response in beech and fir is confirming our former research, showing increasing Φ in beech with increasing light intensity and the opposite, decreasing in fir, respectively (Čater *et al*. 2014). Differences are even more pronounced along the geographical gradient, showing increase in Φ for beech towards the SE in all light categories and decrease in Φ for fir.

Post-hoc analysis confirmed statistically significant differences between all categories of light for both A_max_ and Φ, except on locations with old growth reserves, where no significance has been confirmed between forest edge and open light categories for both beech and fir.

Maximal Φ for beech was observed in open light, for fir under maximal shelter.

When comparing light responses between old growth reserves and the neighbouring managed forests, A_max_ and Φ were in all cases significantly higher than in managed forests. We believe the reason is the microclimate, as the former study within same forest complex highlighted higher relative humidity (RH), higher water use efficiency (WUE) and also photosynthetic nitrogen use efficiency (PNUE) in old growth reserve compared to managed forests in all light categories^37^.

Evident shift of Φ in the edge category towards the open light in all old growth reserves was observed for both species compared to managed forests, while in managed forests the distribution of light categories was more even (Fig. 4).

A_max_ and Φ for both fir and beech confirmed stronger relation between average annual precipitation than between average annual temperature. Relation between Φ and temperature was more pronounced for fir than beech (Table 4).

**Table 4:** Correlation coefficients (r^2^) between average annual precipitation and A_max_ and Φ for beech and fir in all three light categories - in open, edge and under canopy conditions.

			A_max_			Φ		
			r^2^	A	B	r^2^	A	B
Average annual precipitation (mm)	**Beech**	Open	**0.83**	29.29	−0.009	**0.56**	0.032	−0.004
		Edge	**0.76**	31.42	−0.011	0.42	0.029	−0.003
		Canopy	**0.86**	36.54	−0.015	0.40	0.027	−0.004
	**Fir**	Open	**0.83**	39.52	−0.146	**0.57**	0.009	0.006
		Edge	**0.78**	46.67	−0.189	**0.89**	0.008	0.009
		Canopy	**0.86**	54.49	−0.232	**0.86**	0.011	0.009
Average annual air T (°C)	**Beech**	Open	0.39	17.07	−0.006	0.31	0.038	−0.066
		Edge	0.36	16.83	−0.007	0.32	0.037	−0.071
		Canopy	0.36	19.62	−0.010	0.36	0.037	−0.002
	**Fir**	Open	0.33	19.50	−0.084	0.19	0.009	0.086
		Edge	0.44	23.28	−0.124	0.32	0.007	0.133
		Canopy	0.44	29.68	−0.176	0.22	0.011	0.111

A and B parameters for the equation Y = A*exp ^(B*X)^ are presented. Values above 0.50 are marked bold.

### Morphological response

**Figure 5:**
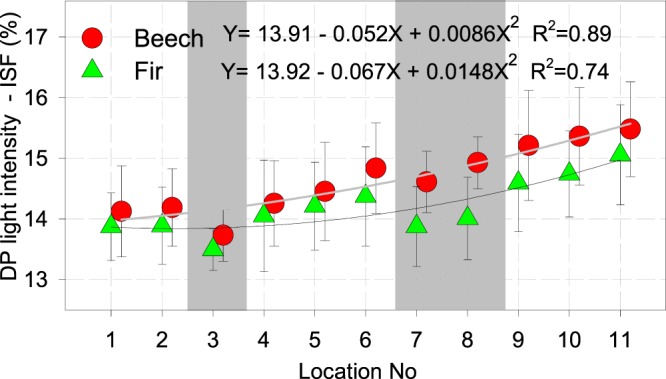
Light intensity at the deflection point (DP). Triggering light intensity (decrease in shade tolerance) increased from NW towards SE (from plot No. 1 towards plot No. 11). Shaded parts represent old growth reserves, bars are standard errors of mean.

Morphological reaction between light intensity and all studied variables (e.g., total openness or direct light) was highest in the case of ISF (data not shown). On all plots, plagiotropic growth was triggered below 17% ISF for beech and fir. Breaking or deflection points (DP), where orthotropic growth changed to plagiotropic growth due to lower light intensity were lowest according to ISF (%) values in old growth reserves - 13.5% for beech, 13.7% for fir, respectively, and increased towards SE (Fig. 5, Table 5). In terms of dispersion of l/h (beech) and 2d/h (fir), the ratio in old growth reserves were in all cases smaller compared to the neighbouring managed sites, while the steepness of curves and maximum declining values were highest, showing bigger shade tolerance.

**Table 5:** Plagiotropic response (dependent) and light intensity ISF (%) (independent variable) for beech and fir: determination coefficients (R^2^), deflection points (DP) expressed in ISF (%), and exponential curve parameters (Y = A + B*exp ^-(C*x)^ for each site (n = 72).

Plot No	Beech					Fir				
	DP	A	B	C	R^2^	DP	A	B	C	R^2^
1	13.9	104.4	1968	0.440	**0.79**	14.1	105.1	387	0.259	**0.78**
2	13.9	103.1	827	0.346	**0.82**	14.2	104.9	385	0.258	**0.76**
3	13.5	103.2	962	0.371	**0.87**	13.7	104.1	419	0.268	**0.80**
4	14.1	103.1	609	0.319	**0.82**	14.3	105.6	690	0.301	**0.79**
5	14.2	103.3	873	0.345	**0.81**	14.5	105.6	606	0.289	**0.72**
6	14.4	102. 0	4016	0.435	**0.85**	14.8	105.5	1567	0.346	**0.88**
7	13.9	102.7	5202	0.475	**0.86**	14.6	105.2	318	0.238	**0.72**
8	14.5	102.3	2647	0.405	**0.84**	14.9	104.7	344	0.236	**0.80**
9	14.6	104.1	592	0.316	**0.83**	15.2	104.3	327	0.225	**0.76**
10	14.9	104.9	693	0.329	**0.78**	15.9	1025	555	0.238	**0.73**
11	15.8	104.8	3867	0.419	**0.89**	16.5	102.9	870	0.259	**0.80**

Plots 3, 7 and 8 delineate old growth reserves.

Relation between DP and precipitation along the studied range was more pronounced for the growing season (R^2^_beech_ = 0.82 and R^2^_fir_ = 0.75) than for the whole year-period (R^2^_beech_ = 0.64 and R^2^_fir_ = 0.67). Stronger relation between the morphological response (DP) and temperature on the whole gradient was confirmed more for the growing period (R^2^_beech_ = 0.36; R^2^_fir_ = 0.26) than for the entire year (R^2^_beech_ = 0.25; R^2^_fir_ = 0.12), respectively.

### Climate-growth response

**Figure 6:**
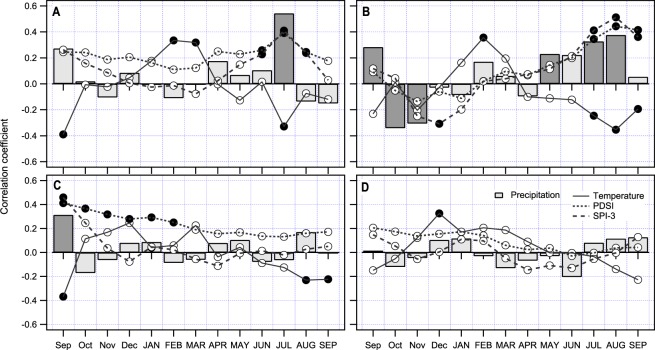
Radial growth response for fir in four selected regions (**A**–**D**) to different climatic parameters - average monthly temperature, monthly sum of precipitation, Palmer Drought Severity Index (PDSI) and 3-month Standardized Precipitation Index (SPI-3). Dark circles and dark bars indicate statistically significant bootstrapped correlations.

Radial growth of fir (Fig. 6) is more sensitive to climate than beech. In regions A and B we confirmed clear summer-precipitation signal, where above average precipitation (*p*) positively influenced radial growth of fir. This was also supported by the positive correlation with 3-month standardised precipitation index (SPI3) and Palmer Drought Severity Index (PDSI), both showing the same direction of correlation as *p*.

Temperature (*T*) influenced the growth of fir in regions A and B, but not to such an extent as *p*. In region A, the correlation values for *p*, SPI3 and PDSI are similar; in region B, correlation between *p* and radial growth decreases, while correlation between SPI3 and PDSI are the same as in region A. We believe these results indicate tree growth as more sensitive to long-term rather than short term water deficit. In more southern regions, C and D, we confirmed only weak correlation between climate variables and fir radial growth. In region C, only PDSI shows correlation between February PDSI (till September of the preceding year) and radial growth. Weak negative correlations between *T* and radial growth in August and September indicate that water required for the growth is accumulated during the winter in a form of snow and slowly released during the growing period. In southernmost region D, no significant correlations between any of the studied climatic variables and radial growth were confirmed. Only two weak correlations between *T*- a positive in December of the preceding year and a negative in September of the year of ring formation were evident. Both were weak and on the edge of significance.

**Figure 7:**
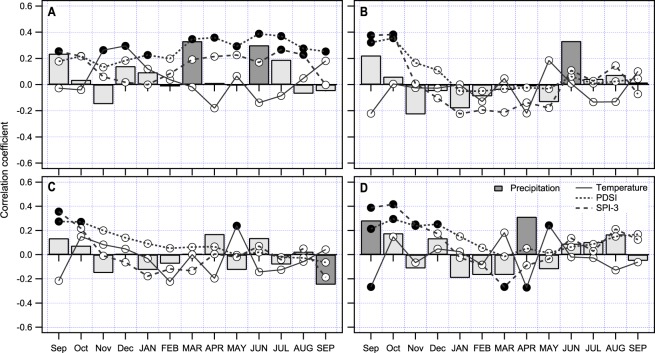
Radial growth response to climate for beech in four selected regions (**A**–**D**) (see Fig. 6).

Growth response of beech (Fig. 7) to climate is different than in fir, although trees from the same locations and forest stands were sampled. In the northernmost region A, beech shows positive response to *p* in March and June as well as significant correlation between drought indices, SPI3 and PDSI and radial growth. PDSI shows more significant correlations, highest in June and July. Response of radial growth to *T* is weak and significant only for November and December of the year prior to ring formation. In region B, significant positive correlation between radial growth and *p* in June was evident. Both drought indices and *T* in the growing period were without significance. In southernmost regions (C and D), growth is characterised by positive response to *T* in May and negative response to *p* in September, which can be associated with abrupt stop of growth. In region D, beech responded positively to April *p* and negatively to above average *T* in April. Relatively high values of correlation with SPI3 in September and October of the year prior to ring formation indicate long-term demand for water, also visible through positive correlation with *p*.

Comparison of tree growth response to climate between managed and old-growth reserves did not confirm any differences; management of studied forests is evidently close-to-natural processes in old-growth reserves, without significant disturbances caused by forest management.

Within the 1901–2010 period, we identified altogether 53 pointer years (PY) for fir and 50 for beech. For fir, 26 PY were positive and 27 were negative; for beech, 27 years were positive and 23 were negative. Number of PY varies between the regions, with B and C having significantly more than regions A and D. We couldn’t find only a single PY year that would be common to all four regions and both tree species. Only one positive PY (1958) was common to beech in all four regions. Several PY were common to two regions, in many cases also for both tree species, for example 1930 was positive for fir and beech in regions B, C and D. Some known PY in Europe, like 2003 and 2006, were even not obvious at all. Hot and dry 2003 is only visible in tree rings of fir in regions B and D, while hot and dry 2006 is visible only in fir in region A. According to number of negative PY, beech seems to be more drought tolerant than fir.

## Discussion

Many studies have predicted substantial changes in forest dynamics during the next century because of GCC. As some spatial distribution models have projected reduction of the distribution areas of fir and beech forests by 2100 owing to climate change to the benefit of more drought-tolerant species^52^, the question of the prevailing impact of ecological as opposed to macroecological and phytogeographical gradients on vegetation is highlighted in many studies^49,53^.

All selected study sites were located above 800 m above sea level; study sites in the SE were located at higher elevations than in the NW. That is understandable, because there is a gradient in T and humidity in the area under study. The mean annual T differed very little among the study sites, showing that despite differences in elevation the study plots represent similar climatic conditions (Fig. 2, Table 1).

According to selection criteria of our studied fir-beech sites, no significant relation was confirmed between measured physiological and morphological parameters and the T, while more pronounced relation was confirmed with *p*, particularly for fir. Change of responses in studied species is in accordance with Marinšek *et al*.^49^, who confirmed increase in proportion of chamaephytes, hemicryptophytes and therotypes towards SE in mesophylous beech forests of SE Europe. We relate comparable amount of nitrogen content in leaves on all plots with similar temperature and site conditions.

Elevations of studied plots above 800 m show constant average and relatively narrow T belt without expressed change in T along the entire geographical gradient. We believe this is the reason for statistically insignificant relation between studied parameters and T or surface solar radiation (both from KNMI database) either during the entire year or during the April-September growing period, respectively. High correlation was confirmed only between locations and different cloud coverage towards SE direction (r^2^ = 0.86), but only during growing period, which could be related with increasing A_max_ values in both species towards the SE and increasingly longer growing period in the SE (Fig. 3). Differences in A_max_ between light categories were significant on all locations and became more pronounced for beech towards SE and in smaller degree for fir (Fig. 3). Φ for beech was highest in the central range (Bosnia and Herzegovina), and for fir in the NW part of the transect (Slovenia, Croatia).

We may relate higher Φ in fir with sites and micro locations with predominating diffuse light and lower Φ in SE with limiting edge of its natural distribution^54^, while Φ for beech culminates on sites with predominately direct light component^42^. Φ on each location for beech was maximal in microsites with maximal light intensity, while for fir it was maximal in shelter, with predominating diffuse light, which is in accordance with our previous work^41,42^. Φ for beech increases with light intensity, so its adaptation ability to light increase is better than in fir. The lower Φ of fir in exploiting high-intensity solar radiation compared to beech may be a competitive disadvantage in large canopy gaps, which could limit species recruitment to the forest understory or small gaps, especially in admixture with beech. It is not clear what caused the shift of Φ in the edge light category in the old growth reserves towards the open category uniformly for both beech and fir, as leaf nitrogen values were comparable between sites and in optimal range on all plots. Beech can tolerate a broad range of understory light levels and manages to recruit in a variety of light conditions in young stages of growth, as it is capable of decurrent and polycyclic growth^55^. Fir is more shade-tolerant than beech, but is also considered a “late-successional species. Its competitive strength is, compared to beech, in low light conditions greater, but in an intermediate and ample light condition it is consequently smaller; in gap-openings, beech adapts better and much faster to rapid changes in light intensity^41,42^, while fir’s adjustment of growth rate to light environment occurs gradually over several years^56^. Competition for light may be more pronounced in old growth reserves compared to managed forests, where larger proportion of forest edge category is artificially created^57^. In old growth reserves, gaps are created after disturbances and present an opportunity for overgrowing of the present tree species.

Morphological adaptation of juvenile trees to various light levels represents an important species-specific characteristic^58^. Relation reflects the control exerted by the apical shoot over the outgrowth of the lateral buds. Plagiotropic growth, usually evident under conditions with lower light intensities and under dense canopies with predominating diffuse light component^44^, may be also influenced by the forest management approach^37^. Beech and firs are better adapted to higher light intensities on sites with applied irregular shelterwood system than on single-tree selection sites, while maximum shade tolerance was confirmed in old growth^37^. Wagner and Müller-Using^59^ quote a limiting value of 10%, while Zang and Biondi *et al*.^60^ state 15% of relative light intensity, below which plagiotropic growth is evident, which accords better with our results.

We believe the selected criteria encompasses the whole plant size and thus responds to the cumulative light environment of the plant during its lifespan and not only the last growing year, as in case of apical dominance ratio (ADR) used by Ripullone *et al*.^61^, who used the ratio between apical shoot length and length of first whorl lateral twigs. There is, however, no clear evidence that shade-tolerant species are morphologically more plastic than less tolerant ones^62^.

Decrease of shade tolerance along the geographical gradient towards SE in both beech and fir is in accordance with Marinšek *et al*.^49^, who confirmed significant increase in ecological indicator values (EIV), especially for light and decrease in EIV for moisture and nutrients for beech forests along the geographical gradient towards SE. The plagiotropic growth responses of trees to different light intensities in our study were non-linear (Table 5); the ratio between canopy density (ISF%) and plagiotropic shape increased exponentially after the light dropped below 13.9% on the greater part of NE plot and below 15.8% on the greater part of SE plot.

In old growth reserves (Fig. 4 - No. 3, 7 and 8), plagiotropic growth in beech and fir was triggered by smaller light intensities (DP) than in managed forests. Assimilation rates (A_max_) and efficiency (Φ) were also higher than on neighbouring managed forest sites.

Fir growth response to climate was slightly stronger than in beech. Both variables - *T* and *p* have stronger influence on growth of fir than on beech. Climate signal in fir diminished from NW to SE, where only drought indices remain significant, while beech response to climate was weaker on all plots and diminished, similar as in fir, from NW to SE.

We confirmed a clear spatial effect in climate response for both species. In northern regions A and B, the response to climate is better than in southern C and D regions, which contradicts our expectations. In southern regions one would expect increasing water deficit, pronounced climate sensitivity and higher correlations with climatic parameters related to precipitation or drought. In two southernmost regions, low climate sensitivity with significant response to above-average temperature and negative response to precipitation (visible in 3-month SPI) in March was confirmed for beech. Similar high values for Slovenia and northern parts of Croatia (e.g. region A) were found by Čufar *et al*.^63^, while similarly low values for the climate-growth relationship for the B, C and D regions were found by Tegel *et al*.^64^ for the sites in Albania and North Macedonia and by Hacket-Pain^65^ for sites in Greece, who confirmed diminishing climate signal in beech after 1990 and increasing growth despite continued dry and hot summer conditions. Low values for the climate growth relationship were also found for old-growth forests^66^ and managed forests^67^ in Bosnia and Herzegovina and for managed forest in Serbia^68,69^. Stjepanović *et al*.^67^, who studied beech growth and climate response in a relatively dense network of beech sites in Serbia and Bosnia & Herzegovina, which correspond to our regions B and C, confirmed that beech responded to climate only at a lower, warmer and drier elevations, and in some cases also at the upper timberline. Beech in the optimal altitudinal distribution range shows weak response to climate and mainly responses to other, non-climatic factors.

Phenomenon of weak to non-existing responses to different climate variables found as we move southwards along the transect were found by many studies^63–68^ and is suggested to be connected to either genetic adaptation, phenotypic acclimation of the species or combination of both^70^. If the results are genetically based, trees within a given location could be much more sensitive to climate change than indicated by the very broad geographic distributions of these temperate tree species, but if the results are phenotypic, this would represent local acclimation that could help buffer species in the face of climate change^70^.

Fir, with higher demand for water than beech, was never regarded as a drought-tolerant species. After a pronounced period of its dieback e.g.^71^, species recovered and shows signs of growth increase^72^. The majority of studies agree that below-average summer T and above-average *p* positively affect its growth e.g.^71–74^. Our study confirms this significant relation in regions A, B and C, but not D. Fir response to climate has a clear spatial element: in NW July *T* and *p* (together with drought indices) were the most influential factors, in region B a wider time frame of the response ranging from May till August for *p*, *T*, PDSI and SPI-3 was confirmed, while in region C the main driving factors are *p* in May and PDSI, indicating importance of water availability at the beginning of the growth. In region D, only PDSI and SPI-3 correlated with growth, confirming again water availability as a major factor influencing the radial growth.

In the middle of the transect, in regions B and C, trees are particularly responsive to extreme years, showing higher number of positive and negative PY compared to regions A and D. We found such response hard to interpret; some studies show that beech might be more vulnerable to drought stress than initially assumed or visible from the measured parameters^75^.

Well preserved condition of studied beech-fir forests is in tight relation with their low management intensity in the past. Their uneven-aged structure above 700 m is similar to the structure of old growth reserves^57^, where elevation represents the key factor controlling the microclimate in temperate mountain forest stands^76^. Liberal selection of felling regimes applied in uneven-aged beech-fir forests, also known as close-to-nature silviculture^77^, employ relatively low intensity and small-scale felling regimes to mimic natural forest composition, structures, and natural disturbances on the lower end of the disturbance severity gradient at stand scales^4,12^. Forest stands managed in such a way generally create stands with small-scale heterogeneous structure and are thought to be both resistant and resilient to disturbance^78^, as was also confirmed by our results. Disadvantages of uneven-aged forestry include the reliance on shade tolerant species, which can be hampered by climatic conditions of open areas created by disturbances.

Further decline of fir may be expected because of its higher proportion in the stand volume than in near-natural forests and by recruitment failure due to overbrowsing and strong loss of vitality^79^. Limited silvicultural options for preserving fir in an adequate proportion in mixed mountain forests, where both climate change and browsing pressure are present, may lead to a non-compensatory effect, where increase in browsing pressure may enhance the shift in dominance of tree species^80^.

Different response in beech and fir is confirming our former research^42,57^, showing increasing efficiency in beech with increasing light intensity and the opposite, decreasing in fir, respectively^42^. Physiological and morphological differences are even more pronounced along the geographical gradient, showing efficiency increase in beech towards the SE in all light categories and decrease in fir. Morphologic changes indicate reduced shade tolerance towards SE for both species. Reasons for such response might be in the natural range of the species distribution or better plasticity in beech compared to fir.

Fir in the SE part reaches its southernmost range of natural distribution, where droughts and precipitation deficit are more pronounced compared to the NW region of the study.

## Conclusions

**Figure 8:**
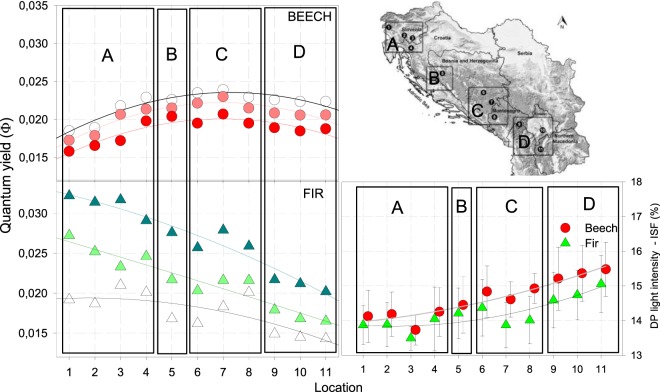
Physiologic and morphologic responses within growth areas (**A**–**D**) along the studied gradient.

Comparison of the two consecutive referential periods 1995–2004 and 2005–2015 within the April-September growing period confirmed a drop of total *p* and a rise of average annual air T on the studied geographical transect. According to the observed efficiency of both species, it is reasonable to expect increased dominance of beech over fir in the regeneration phase. Physiological and morphological response observed during three consecutive years confirmed good agreement with long term growth response (Fig. 8). Reduced shade tolerance and higher efficiency of beech in the south may confirm current boost in regeneration of beech also in the old growth forests and could indicate better adaptation of forest stands with diversified microstructure and bigger share of forest gaps. Emphasis should be given to sites under mature canopy light conditions where fir outcompetes beech to preserve and increase its share in regeneration phase.

Our findings add evidences of divergent tree response in the Mediterranean basin and show a gradual transition between forests where positive (temperate) and negative (Mediterranean) growth trends dominate. We believe that preservation of uneven-aged structure, emphasis on fir regeneration and reduction of ungulates present key steps for further stability in those close-to-nature mixed forest ecosystems.
